# Prolonged Emergency Department Length of Stay as a Predictor of Adverse Outcomes in Patients with Intracranial Hemorrhage

**DOI:** 10.1155/2015/526319

**Published:** 2015-01-01

**Authors:** Erica M. Jones, Amelia K. Boehme, Aimee Aysenne, Tiffany Chang, Karen C. Albright, Christopher Burns, T. Mark Beasley, Sheryl Martin-Schild

**Affiliations:** 1 Comprehensive Stroke Center, Department of Neurology, Tulane University, New Orleans, LA, USA; 2 Department of Neurology, Columbia University, New York, NY, USA; 3 Departments of Neurosurgery and Neurology, University of Texas Medical School at Houston, USA; 4 Department of Epidemiology, School of Public Health, University of Alabama at Birmingham, Birmingham, AL, USA; 5 Department of Biostatistics, School of Public Health, University of Alabama at Birmingham, Birmingham, AL, USA

## Abstract

**Objectives:**

Extended time in the emergency department (ED) has been related to adverse outcomes among stroke patients. We examined the associations of ED nursing shift change (SC) and length of stay in the ED with outcomes in patients with intracerebral hemorrhage (ICH).

**Methods:**

Data were collected on all spontaneous ICH patients admitted to our stroke center from 7/1/08–6/30/12. Outcomes (frequency of pneumonia, modified Rankin Scale (mRS) score at discharge, NIHSS score at discharge, and mortality rate) were compared based on shift change experience and length of stay (LOS) dichotomized at 5 hours after arrival.

**Results:**

Of the 162 patients included, 60 (37.0%) were present in the ED during a SC. The frequency of pneumonia was similar in the two groups. Exposure to an ED SC was not a significant independent predictor of any outcome. LOS in the ED ≥5 hours was a significant independent predictor of discharge mRS 4–6 (OR 3.638, 95% CI 1.531–8.645, and *P* = 0.0034) and discharge NIHSS (OR 3.049, 95% CI 1.491–6.236, and *P* = 0.0023) but not death.

**Conclusions:**

Our study found no association between nursing SC and adverse outcome in patients with ICH but confirms the prior finding of worsened outcome after prolonged length of stay in the ED.

## 1. Introduction

The delivery of acute stroke care in the emergency department (ED) prior to admitting a patient to the stroke unit is of utmost importance. In order to reduce the delay in early acute evaluation and management ED teams are trained to implement the National Institute of Neurological Disorders and Stroke guidelines [1]. For critically ill patients, such as those suffering from intracerebral hemorrhage (ICH), the initial care provided in the emergency department (ED) impacts patient outcomes.

Approximately 10–30% of all strokes are of the hemorrhagic type, with limited evidence based treatment options [2]. Monitoring of vital signs, level of consciousness, and neurological condition by ED nurses during the early phase of ICH treatment and monitoring is crucial. Prior work has shown a relationship between increased ED length of stay (LOS) and poor outcomes of patients with cerebrovascular disease [3, 4]. In a study assessing outcomes after acute ischemic stroke (AIS), patients that were present in the ED during a nursing shift change (SC) were at 2.5 times the odds of developing pneumonia [4]. The longer a patient spends in the ED, the more likely they are to experience a SC. Inadequate communication during a handoff at SC can lead to medical errors and worse patient outcomes [5]. This study explores the association between ED nursing SC and ED LOS on the outcomes of patients with ICH.

## 2. Methods

All stroke patients at our institution are prospectively enrolled in a stroke registry during their stroke admission. We performed a retrospective, observational study of prospectively enrolled stroke patients presenting from July 2008 to June 2012 with ICH to the ED of our Comprehensive Stroke Center. Patients who were admitted to the ED and had complete data for the times of admission and discharge from the ED were included. Patients who were transferred from another hospital, suffered in-hospital ICH, or had primary intraventricular hemorrhage (IVH) were excluded. The ED physicians are not involved in the care of stroke patients once they are admitted to the stroke service. ED nurses, stroke service resident physicians and/or nurse practitioners, and a stroke service attending physician are the only providers involved in the care of admitted patients with stroke in the ED. The ED nurses change shift twice daily between 07:00 to 08:00 and 19:00 to 20:00. The nursing SC is a verbal report, given near the patient’s bedside, but does not include formal review of stroke-specific orders, such as head-of-bed positioning, status of dysphagia screening, and/or speech therapy recommendations for diet.

Patients were grouped based on presence during ED nurses SC (i.e., 07:00–08:00 and 19:00–20:00) and patients were categorized as being in the ED for <5 hours and ≥5 hours [3, 4]. Previous research in this subject has used 5 hours as the time cut-off. In order to be generalizable to other studies on this subject we used what has been previously used in the literature. This study was approved for expedited review by our Institutional Review Board. Details of our institution, population, and protocol have been previously published as well as the data on the methods for collection of patient data into our stroke registry [4, 6].

Short-term outcomes were favorable discharge disposition (discharge to home or inpatient rehabilitation center), discharge National Institute of Health Stroke Scale (NIHSS), and discharge modified Rankin Scale (mRS) score. Poor functional outcome was defined as discharge mRS 4–6. Neurological deterioration was defined as an increase in NIHSS score of 2 or more points within a 24-hour period [7]. Discharge NIHSS was divided into three categories since it was shown to be a strong predictor of mortality as a continuous or categorical variable [8].

Baseline characteristics were assessed using Wilcoxon rank sum tests and chi-square tests. Length of stay ED (LOS) was dichotomized into <5 hours and ≥5 hours [3, 4]. Crude and adjusted logistic regression and cumulative logit models were used to determine if SC or ED LOS was significant independent predictor of short-term outcomes, adjusting for baseline ICH score [9], which includes evaluation of age, GCS, ICH volume, ICH location, and presence or absence of IVH.

## 3. Results

A total of 162 patients met the inclusion criteria for the study, of which 60 (37.0%) were present in the ED during a nursing SC. ICH score was not significantly different between groups (Table 1). Patients present in ED during a SC spent significantly more time in the ED (306 versus 218 minutes; *P* = 0.0007) and had a lower stroke severity as measured by NIHSS (14 versus 17; 0.0446). Intubation and pneumonia rates were similar between groups.

Nursing SC was not a significant predictor of favorable discharge disposition (OR 1.29, 95% CI 0.57–2.90, and *P* = 0.54), poor functional outcome (OR 0.75, 95% CI 0.33–1.72, and *P* = 0.49), discharge NIHSS (OR 0.94, 95% CI 0.48–1.84, and *P* = 0.85), nor mortality (OR 1.04, 95% CI 0.41–2.62, and *P* = 0.94). After adjusting for ICH score, intubation was a significant predictor of poor discharge disposition (OR 5.60, 95% CI 2.19–14.5, and *P* = 0.0003) and death (OR 4.88, 95% CI 1.76–13.5, and *P* = 0.0023) but not pneumonia (OR 1.87, 95% CI 0.64–5.47, and *P* = 0.25).

Table 2 shows the baseline characteristics stratified by ED LOS. The median ED LOS was 268 minutes (range 51–2944). ED LOS was at least 5 hours in 56 (34.6%) patients. Patients who were in the ED for longer than 5 hours were older (65 versus 59; *P* = 0.0265) and had lower NIHSS on admission (15 versus 16; 0.06). In the unadjusted models longer ED LOS was a significant predictor of poor functional outcome (OR 2.89, 95% CI 1.34–6.22, and *P* = 0.0067) and discharge NIHSS (OR 2.07, 95% CI 1.12–3.83, and *P* = 0.0201) but not of poor discharge disposition (OR 1.38, 95% CI 0.72–2.67, and *P* = 0.34) or death (OR 0.97, 95% CI 0.45–2.09, and *P* = 0.93). In the adjusted models, longer ED LOS was a significant predictor of poor functional outcome (OR = 3.64, 95% CI 1.53–8.65, and *P* = 0.0034) and discharge NIHSS (OR 3.05, 95% CI 1.49–6.24, and *P* = 0.0023), but not of discharge disposition (OR = 1.89, 95% CI 0.81–4.42, and *P* = 0.14), or death (OR = 1.32, 95% CI 0.51–3.40, and *P* = 0.56) after adjusting for ICH score at baseline.

## 4. Discussion

Our study found that an ED nursing shift change was not associated with poor outcomes in patients presenting with ICH. While we found no adverse outcome to be associated with ED nursing SC, we found that those patients who spent more than five hours in the ED were more likely to experience a shift change and experienced worse functional outcome regardless of incoming ICH score. Our findings are consistent with previous work that demonstrated critically ill stroke patients who spent more than 5 hours in the ED had a 4-fold increase in the odds of poor outcome, but about half of these patients had ischemic stroke, while our sample was limited to patients with spontaneous ICH [3]. However, a recent study on outcomes in the ICH population showed no effect of ED LOS on outcome after controlling disease severity, but a large proportion of their patient population was transferred from an outside hospital [10]. In our study we excluded transfer patients and determined that increased ED LOS was associated with an almost 4-fold increased odds of worse functional outcomes and a 3-fold increased odds of worse discharge NIHSS score.

Our prior findings from investigation of the association of ED shift change and LOS in acute ischemic stroke patients found higher frequency of pneumonia in patients present during shift change and with ED LOS at least 5 hours, which is associated with worse outcome [4]. The rate of pneumonia and intubation in ED was similar comparing SC and ED length of stay groups in our ICH population. Our study does illustrate that intubation of the ICH patient is related to worse outcomes, regardless of ED shift change or LOS. Potential explanations for the difference outcome based on ED LOS include deficits in critical care provided in the ED relative to the neurological ICU (NICU) or loss of advanced neurology intensivist care provided in NICU during the early critical hours after onset. Because the relationship between ED LOS and functional outcome persisted after adjustment for ICH score, the preferential transfer to the ICU for patients more likely to survive is unlikely to explain our findings. Further study is warranted to determine the impact of SC and ED LOS on other risk factors for poor outcome in patients with ICH.

Our study has several important limitations. This is a single center, retrospective, and cross-sectional design limited by the information in medical records. Relevant information on SC procedures and hospital bed allocation processes may not be ascertainable. The retrospective assessment of the mRS score for some patients may limit the validity of this outcome measure, but mRS scores were determined blinded to the exposure of the SC and prior to development of the research question. Our single center experience makes generalizability somewhat limited. We also did not analyze time from symptom onset to ED arrival, which may have predicted hematoma expansion and neurological deterioration in the ED, predictive of higher mortality and worse neurologic outcomes [11]. To the best of our knowledge, this is the largest reported study of time in the ED and SC in ischemic stroke patients. However, the small sample size may be limiting our ability to detect differences, should they exist, especially in subset analyses.

This study highlights the relationship between prolonged time in the ED and increased odds of poor functional outcome and discharge NIHSS in patients presenting to the ED with ICH. In our center, ICH patients who present during an ED shift change are not at increased odds of poor outcomes. Further research is needed to assess if prolonged time in the ED is associated with increased risk of poor outcomes for ICH patients in other centers.

## Figures and Tables

**Table 1:** Baseline demographic and clinical characteristics stratified by shift change.

Variables	No shift change *N* = 102	Shift change *N* = 60	*P* value
Age, median (range)	60 (19–92)	63 (29–87)	0.13
Gender, % male	52 (50.9%)	37 (61.7%)	0.19
Baseline NIHSS, median (range)	17 (0–40)	14 (0–40)	0.0446
Baseline GCS, median (range)	14 (3–15)	14 (3–15)	0.36
Baseline ICH score, median (range)	2 (0–5)	1 (0–5)	0.21
Glucose, median (range)	135 (70–421)	133 (85–329)	0.81
Length of stay in ED in minutes, median (range)	218 (51–739)	306 (68–2944)	0.0007
Intubated in the ED, %	33 (32.3%)	18 (30%)	0.76
Intubated by EMS, %	3 (2.9%)	1 (1.7%)	0.37
Neuroworsening, %	59/84 (70.2%)	30/48 (62.5%)	0.36
Pneumonia, %	17 (16.8%)	10 (16.7%)	0.98
Length of stay in days, median (range)	7 (1–86)	9 (1–34)	0.93
mRS on discharge, median (range)	4 (0–6)	4 (1–6)	0.31
Discharge mRS percents			
mRS 0–2, %	12 (11.8%)	8 (13.3%)	0.77
mRS 3–4, %	42 (41.2%)	29 (48.3%)	0.38
mRS 5–6, %	48 (47.1%)	23 (38.3%)	0.28
Discharge NIHSS, median (range)	14 (0–42)	11 (0–42)	0.42
Discharge NIHSS			
NIHSS 0–6, %	32 (32.3%)	22 (37.3%)	0.52
NIHSS 7–22, %	34 (34.3%)	21 (35.6%)	0.87
NIHSS >22, %	33 (33.3%)	16 (27.1%)	0.41
Disposition, %			0.80
AMA	0	0	
Home or home with home health	17 (16.7%)	11 (18.3%)	
Inpatient rehab	39 (38.2%)	23 (38.3%)	
Transfer to another service	1 (0.98%)	0	
SNF/NH	12 (11.8%)	7 (11.7%)	
LTAC	4 (3.9%)	5 (8.3%)	
Hospice	5 (4.9%)	1 (1.7%)	
Death	24 (23.5%)	13 (21.7%)	
Good disposition (home or inpatient rehab), %	56 (54.9%)	34 (56.7%)	0.83
Death, %	24 (23.5%)	13 (21.7%)	0.78

**Table 2:** Baseline demographic and clinical characteristics stratified by ED length of stay.

Variables	ED LOS < 5hrs *N* = 106	ED LOS ≥ 5hrs *N* = 56	*P* value
Age, median (range)	59 (19–92)	65 (25–92)	0.0265
Gender, % male	63 (59.4%)	26 (46.4%)	0.11
Baseline NIHSS, median (range)	16 (0–39)	15 (0–40)	0.06
Baseline GCS, median (range)	13 (3–15)	14 (3–15)	0.58
Baseline ICH score, median (range)	1 (0–5)	1 (0–5)	0.79
Glucose, median (range)	131 (70–421)	138 (84–329)	0.78
Intubated in the ED, %	37 (34.9%)	14 (25%)	0.20
Intubated by EMS, %	2 (1.9%)	2 (3.6%)	0.31
Neuroworsening, %	60/86 (69.8%)	29/46 (63%)	0.43
Pneumonia, %	18 (17.4%)	9 (16.1%)	0.86
Length of stay in days, median (range)	9 (1–50)	5 (1–86)	0.0154
mRS on discharge, median (range)	4 (0–6)	4 (0–6)	0.13
Discharge mRS percents			
mRS 0–2, %	10 (9.4%)	10 (17.9%)	0.12
mRS 3–4, %	47 (44.3%)	24 (42.9%)	0.86
mRS 5–6, %	49 (46.2%)	22 (39.3%)	0.40
Discharge NIHSS, median (range)	15 (0–42)	6 (0–42)	0.0382
Discharge NIHSS			
NIHSS 0–6, %	25 (24.3%)	29 (52.7%)	0.0003
NIHSS 7–22, %	46 (44.7%)	9 (16.4%)	0.0004
NIHSS >22, %	32 (31.1%)	17 (30.9%)	0.98
Disposition, %			0.16
AMA	0	0	
Home or home with home health	12 (11.3%)	16 (28.6%)	
Inpatient rehab	44 (41.5%)	18 (32.1%)	
Transfer to another service	1 (0.94)	0	
SNF/NH	15 (14.1%)	4 (7.1%)	
LTAC	6 (5.7%)	3 (5.6%)	
Hospice	4 (3.8%)	2 (3.6%)	
Death	24 (22.6%)	13 (23.2%)	
Good disposition (home or inpatient rehabilitation center), %	56 (53.8%)	34 (60.7%)	0.34
Death, %	24 (22.6%)	13 (23.2%)	0.93
